# The production of fuels and chemicals in the new world: critical analysis of the choice between crude oil and biomass vis-à-vis sustainability and the environment

**DOI:** 10.1007/s10098-020-01945-5

**Published:** 2020-09-21

**Authors:** Vikramaditya G. Yadav, Ganapati D. Yadav, Saurabh C. Patankar

**Affiliations:** 1 Department of Chemical and Biological Engineering, The University of British Columbia, Vancouver, BC Canada; 2 School of Biomedical Engineering, The University of British Columbia, Vancouver, BC Canada; 3 Department of Chemical Engineering, Institute of Chemical Technology, Mumbai, 400019 India; 4 Department of Chemical Engineering, Institute of Chemical Technology Mumbai, Indian Oil Odisha Campus, Bhubaneshwar, India

**Keywords:** Biomass, Petroleum, Shale oil and gas, Valorization, Energy, Environment, Sustainability, Hydrogen economy, Methanol economy, Methane, Renewable sources

## Abstract

**Abstract:**

Energy and the environment are intimately related and hotly debated issues. Today’s crude oil-based economy for the manufacture of fuels, chemicals and materials will not have a sustainable future. The over-use of oil products has done a great damage to the environment. Faced with the twin challenges of sustaining socioeconomic development and shrinking the environmental footprint of chemicals and fuel manufacturing, a major emphasis is on either converting biomass into low-value, high-volume biofuels or refining it into a wide spectrum of products. Using carbon for fuel is a flawed approach and unlikely to achieve any nation’s socioeconomic or environmental targets. Biomass is chemically and geographically incompatible with the existing refining and pipeline infrastructure, and biorefining and biofuels production in their current forms will not achieve economies of scale in most nations. Synergistic use of crude oil, biomass, and shale gas to produce fuels, value-added chemicals, and commodity chemicals, respectively, can continue for some time. However, carbon should not be used as a source of fuel or energy but be valorized to other products. In controlling CO_2_ emissions, hydrogen will play a critical role. Hydrogen is best suited for converting waste biomass and carbon dioxide emanated from different sources, whether it be fossil fuel-derived carbon or biomass-derived carbon, into fuels and chemicals as well as it will also lead, on its own as energy source, to the carbon negative scenario in conjunction with other renewable non-carbon sources. This new paradigm for production of fuels and chemicals not only offers the greatest monetization potential for biomass and shale gas, but it could also scale down output and improve the atom and energy economies of oil refineries. We have also highlighted the technology gaps with the intention to drive R&D in these directions. We believe  this article will generate a considerable debate in energy sector and lead to better energy and material policy across the world.

**Graphic abstract:**

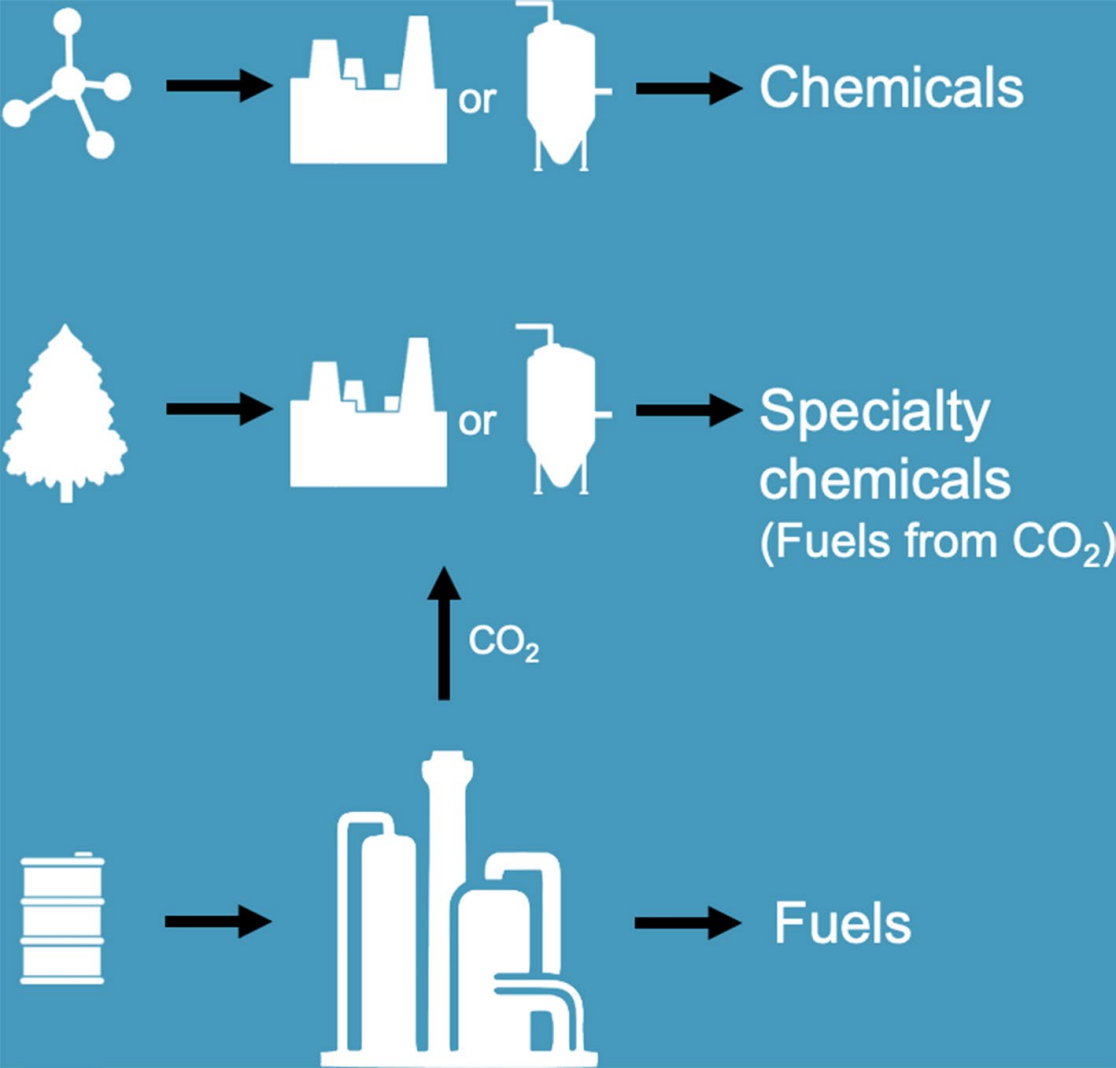

**Electronic supplementary material:**

The online version of this article (10.1007/s10098-020-01945-5) contains supplementary material, which is available to authorized users.

## Introduction

So abruptly, due to the COVID-19 pandemic, the energy scenario across the globe has become topsy-turvy. In our wildest dream in April 2020, we would not have imagined the so-called negative price for crude oil. Bafflingly, it happened and slowly recovered to another low. Brent crude oil spot prices averaged $18/bbl in April 2020, reaching in July–August 2020, ~ $43/bbl on average (US Energy Information Administration 2020). Unless the oil price rises to ~ $45/bbl, the industry will not be viable as some pundits predict. The price war between Saudi Arabia and Russia had already brought down the prices substantially during past few months and COVID-19 pandemic further affected the spot price. The oil supply and demand has been analyzed by the US Energy Information Administration (EIA) based on lockdown in several countries, restrictions on travel, absence of sporting activities, loss of business and recession in many industries, among other reasons. Such is the impact of COVID-19 on the global economy that it has resulted in to both supply and demand for global goods to a new low because as manufacturing activities are shuttered, workers are made redundant, and employers are compelled either to reduce their businesses drastically or close totally, adding to the woes of many. Debates on global recession, or even depression are discussed in the media. The fracking business is affected to the level of extinction and seven of the most active fracking companies in Texas have already cut $7.6 billion from their budgets as a response to the collapse in oil prices (Jamail 2020). Although the predictions of the US Energy Information Administration are somewhat hazy, the restrictions on travel may be relaxed or partially lifted by the fourth quarter of 2020 and/or until such time a vaccine or therapeutic is available across the world. Even otherwise lockdown will be lifted with new ways of lifestyle and movement. EIA has predicted that Brent crude oil prices will increase to ~ $32/bbl during the second half of 2020 reaching $54/bbl by the end of 2021, which is based on an expected worldwide consumption to 94.22 MMBD during the second half of 2020 (US Energy Information Administration 2020). It is also mentioned by EIA that the US shale industry will play a role in this price structure. Some of our arguments in this article consider the short-term effect of COVID-19 as an aberration and more importantly the post-COVID 19 scenario as a long-term strategy.

Notwithstanding these current issues, the post-pandemic world will have to adopt a new order. The sustainable production of fuels, chemicals and materials without affecting the environment will be discussed in what follows. Besides, the role of the hydrogen economy, which some countries are talking about and soon might be a reality, will be covered. We have also attempted to critically analyze the historical development of the oil industry and presented a perspective which will attract a wider debate. We have omitted any discussion on renewable energy resources such as solar, wind, geothermal, and tidal, the other non-carbon sources. Hydrogen has a role in carbon economy based on crude oil, coal or biomass. We have deliberately given a brief account of rise of the oil industry to juxtapose its relevance with the bio-based manufacture of fuels and chemicals in the longer run.

## The carbon-based economy

**Fig.1:**
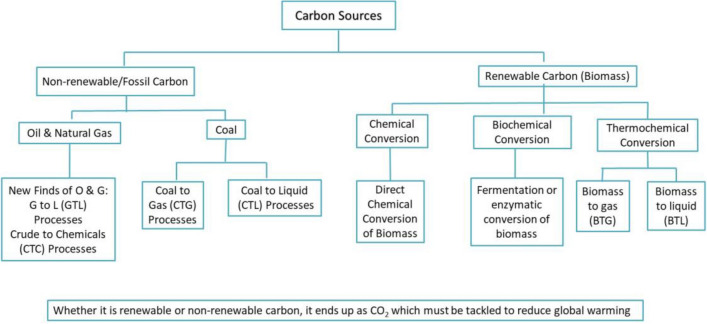
Carbon conversion processes to manufacture useful products. Carbon has been solely responsible for advancement in lifestyle, comfort, luxury, transport, instant communication and longevity

For over past three centuries, carbon in the form of fossils has been the major source of energy and organic fuels, chemicals and materials including polymers. Whether it is the fossil carbon or the renewable carbon the human civilization has advanced due to the conversion of carbon in to a variety of products using well-developed processes, which have greatly added to the modern means of luxury, comfort, transport, communication and longevity (Fig. 1).

The birth of organic chemical industry can be traced to William Perkin’s original work on the synthetic dye mauvine in 1856 which led to great many strides thence and the population growth took a steep upward path leading to more use of carbon as a fuel and then the industrial growth for the production of different of chemicals, solvents, pharmaceuticals, synthetic materials and polymers and communication (Aftalion 2001). The story of coal as a feedstock to chemical production began in Germany in early 1900s with major chemical empires but coal was slowly replaced in 1920s and 1930s by crude oil-based economy. WWII was an incentive for the oil industry in the search for technological advances in refining processes; especially military aviation necessitated huge quantities of high-octane gasoline. The refinery industry, especially the Allied Powers side, having access to large quantities of oil, dedicated its best R & D, talents and resources to respond to the needs of war. From the laboratories of the oil and technology companies, as well as from the academia, path-breaking processes such as alkylation, isomerization, toluene production for explosives were launched and, finally, the greatest innovation among the processes of conversion of heavy fractions of oil, catalytic cracking into fluidized-bed catalytic cracking (FCC) technologies was the pinnacle (Larraz 2019).The superior quality of aviation fuel due to innovative catalytic technologies was responsible for much higher speeds of warplanes during WW II and is the major factor for victory of the Allied Powers as the history records it (Cooling 1994).

Coal was soon forgotten for decades as feedstock for chemical production due to its pollution related issues and complex plants, and abundance of cheap oil. However, coal use has risen from 25% to almost 30% of world energy use, particularly in China. As standards of living increase in developing nations coal use can only increase (Helm 2102; BP Energy 2020). However, due to different reasons the coal to chemicals industry is revived in some part of the world led by China (Speight 2016). Due to abundant coal reserves and its lack of oil, China has invested in the clean coal chemical technologies to produce petrochemical products such as olefins, ethylene and propylenes^.^ Coal-to-liquid (CTL) plants for making synthetic fuels or aromatics and other chemicals, and Coal-to-Substitute Natural Gas (CTSNG) plants for producing methane wherein coal gasification technologies are used to gasify coal with oxygen to produce syngas, which it then transformed into fuels or other chemicals, using technologies like Fischer Tropsch. Coal-to-Olefins (CTO) investments will be challenging if low crude oil pricing persists. The investment economics in China for Methanol-to-Olefins (MTO) and propane dehydrogenation (PDH) are unviable under current spreads among methanol, LPG and olefins (Xie et al. 2010). However, these plants are high emitters of CO_2_ which need to be tackled by using the concept of CO_2_ refineries which is discussed separately.

No matter what source of carbon we use, the life cycle shows its ends up as CO_2_. Unless technologies are developed to mitigate CO_2_ the debate on the source of fuel will continue. In this regard, a historical perspective is provided in this article about the future of oil industry vis-à-vis biorefinery and the role of hydrogen in protecting the environment.

## The emergence of oil refining industry and its global impact

**Fig.2:**
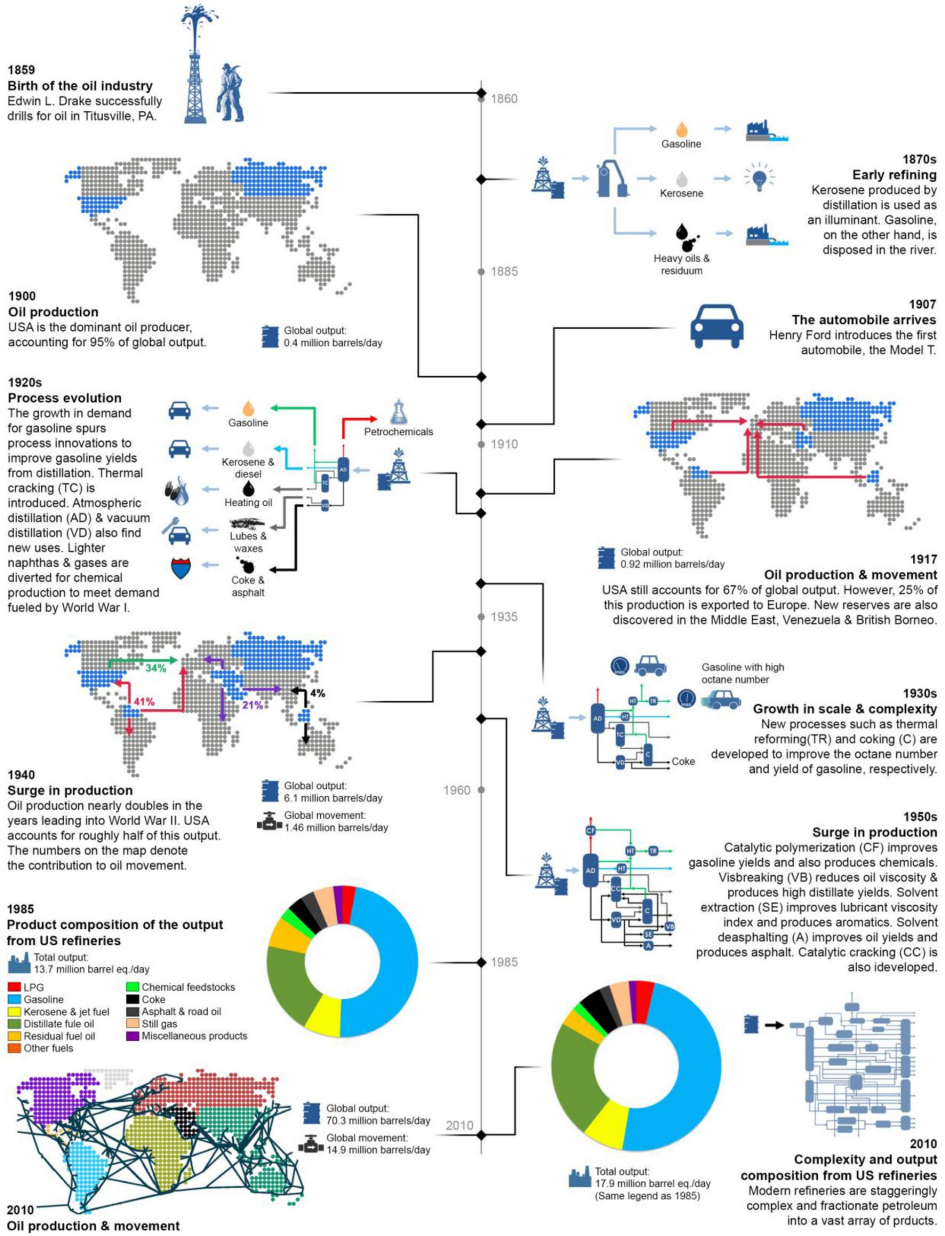
The roughly 160 year history of the oil industry can be summarized as a quest for high atom and energy economies, and wider crack spreads

Few novels capture the zeitgeist of their times as accurately as the story of Captain Ahab’s frenetic and maniacal quest to kill Moby Dick (Melville 1922). Herman Melville’s *Moby Dick* explores complex issues such as class, human nature and spirituality, and its unique narrative style has ensured its special place in American and world canon. Yet, Moby Dick goes much beyond being a literary classic. Published a few years prior to the Pennsylvania Oil Rush, the novel is a timeless allegory of technological tipping points that hasten the rise and fall of industries—in this case, the whale oil industry in North America. Figure 2 gives a pictorial snapshot of roughly 160 year history of the oil industry which can be summarized as a quest for high atom and energy economies, and wider crack spread. The crack spread is defined as the difference between the price of a barrel of crude oil and the cumulative price of all petroleum products that are refined from it, and the metric is an approximation of a refinery’s margins, and the wider the spread, the more profitable the refinery is. Important milestones happened from the beginning in 1859, major events happened in 1907 (birth of automobile industry), 1920 (process evolution), 1940s, 1950s (surge in production, cat cracking), 1985 (many products) and 2010 (increased complexity). Refineries increased utilization of every carbon atom.

At the turn of the eighteenth century, the use of whale oil (the original bioresource may amuse some in today’s context) as an illuminant was slowly taking root in New England and a rudimentary supply chain had been established to meet demand. Whales were hunted off the New England coast and their carcasses were shipped back to nearby ports, where the oil-rich blubber was isolated. In what was one of the earliest successful demonstrations of distillation, the whale oil was subsequently produced by heating the blubber in huge stills at ‘oileries’ located at the ports (Tertzakian 2007). Nevertheless, spoilage of the carcasses significantly reduced the atom and energy economies of whale oil production. To constrain these losses and drive margins higher, whalers began commissioning larger vessels and equipping them with onboard stills in order to produce whale oil on the ships themselves. Onboard distillation of blubber improved oil yields and made the supply chain leaner, which greatly reduced the time it took for whale oil to reach the market. In due course, as the scale of whaling expanded, whale oil also became an important feedstock for the manufacture of soaps and cosmetics. As the nineteenth century dawned, North America was in the midst of a whale oil boom, and whaling corporations had become the super majors of their time.

The demand for domestic illuminants continued to rise unabatedly throughout the nineteenth century, and frequent technological improvements in whaling and refining ensured that the need for whale oil continued to be filled for much of this period. However, by the 1830s, after nearly a century and a half of being relentlessly hunted, whale populations in the Atlantic had been decimated to the point of extinction. Whaling ships were now making longer and more arduous voyages in search of elusive game, and the supply chain for whale oil was coming apart at its seams. The growing energy crunch created an urgent need to replace whale oil with more sustainable alternatives.

Kerosene, a clear liquid that could be produced by distilling either coal or crude oil, emerged as an early favorite on account of its superior luminosity. However, producing kerosene at the levels required to meet the demand for illuminants proved to be a steep challenge. Although coal was plentiful at the time, distilling it to kerosene was quite uneconomical. On the other hand, while crude oil offered a more economical route, it was not as abundant as coal. Crude oil had hitherto only been observed in coal mines and salt wells in Pennsylvania, New York and Ontario, and that too as seepage in nearby low-lying or dug places. Sensing a lucrative business opportunity in supplying kerosene to the masses, several North American entrepreneurs established oil prospection ventures to identify more bountiful sources of crude oil (Yergin 1990). Chief amongst them was Edwin L. Drake, who focused his energies on discovering oil deposits in and around Titusville, Pennsylvania. In August 1859, Drake finally struck oil, setting off the Pennsylvania Oil Rush in the process. The energy industry—and civilization, for that matter—was about to have a paradigm shift (Remsberg and Higdon 1994).

In little over 160 years since, oil refining has evolved from the use of solitary stills for fractionating crude oil into products such as kerosene to the complex, integrated petrochemical refineries that dot distant corners of the globe (Fig. 2). Such is the scale of refining today that the United States alone consumed an average of ~ 20.46 MBPD of petroleum or a total of about 7.47 billion barrels of petroleum products in 2019 (US Energy Information Administration 2020), which translates to a whopping 236 -barrel equivalents per second (bps).

Global output is over 4 times greater, and the quantity of oil transiting around the world in pipelines and on tankers nearly equals the entire refinery output of the United States. Pre-COVID-19, the global production of crude oil was ~ 100.5 MMBD, which slipped to ~ 82 MMBD about mid-April 2020. The demand for fuel went down dramatically because of lock-down in over 100 countries. Significantly, since the prices of a refinery’s inputs and outputs are highly volatile and significantly impacted by demand and supply dynamics (Canadian Fuels Association 2013; US Energy Information Administration 2020), refiners have been perennially optimizing their operations in order to improve their atom and energy efficiencies, thereby maximizing the ratio of their outputs to inputs, as well as their ‘crack spreads’. Since the composition of crude oil greatly impacts the distribution of products that can be refined from it, which, in turn, can profoundly alter the crack spread, refineries have become larger, more complex, more integrated and more flexible over the years in order to process a wider range of crude oils with minimal impact on their crack spreads.

Altogether, the configuration and complexity of a refinery, and its location and proximity to transportation infrastructure can widen the crack spread by as much as $10/bbl (Canadian Fuels Association 2013), and these parameters have been optimized aggressively over the course of the past century to maximize profits. The price of crude oil and its rate of production has always been a matter of geopolitics. Impressive as this technological transformation has been, the role of crude oil in the evolution of modern refinery cannot be understated. Its high energy density and recalcitrance to degradation facilitate its transportation across great distances. These characteristics have greatly shaped our refining infrastructure. Additionally, crude oil is largely fungible, which allows refineries to adapt to market vacillations more efficiently.

Of late many refiners are thinking on the lines of crude to chemicals (C2C) business because by using refinery technology in petrochemical operations extends the scope for the optimization of catalyst processing and energy savings which is a result of the feedstock cycle leading to more profit (Shell 2020). Indeed, many petrochemicals are manufactured as side streams during crude oil refining, of which primary goal remains transportation fuel production. While most refineries convert ~ 5–20% of crude into petrochemicals, some existing refineries have 45% of the output as chemicals, including olefins, aromatics, glycols, and polymers. When more of renewable energy will be used for energy, more and more chemicals be produced going to 60–80%. In other words, variable capacity refineries will be designed in the future. To improve flexibility, it would be better to directly crack crude oil to produce chemicals, particularly light olefins (C2, C3 and C4), using technologies derived from FCC (Corma et al. 2017). New refineries are planned with a variable fuel to petrochemical ratio in view of the less demand for fuel due to ever growing contribution of renewable energy, which is coupled with growing demand for petrochemicals and their use in subsequent tertiary and quaternary industry.

## The quest for sustainable alternatives to crude oil

Like the whale-oil industry a century and six decades ago, though, the petroleum industry now finds itself at a tipping point. The over-use of refined products has wreaked considerable damage on the environment, and the industry has been vilified by all and sundry for its role in climate change (Hamilton 1998; Intergovernmental Panel on Climate Change 2014). There is now a feverish movement to mitigate the industry’s footprint and replace crude oil with more sustainable alternatives to meet the expanding needs of consumer driven economies. Among the candidates considered, biomass has emerged as an early favorite. Renewable energy systems include wind power, biomass, photovoltaics, hydropower, solar thermal, thermal ponds, and biogas. All renewable energy systems must be considered because only about 40 years of oil and gas reserves remaining, in addition to 50–100 years of coal reserves (Pimentel 2008). Biomass, ranging from purposely grown energy crops, wood or forest residues, agri-waste, etc. is considered attractive as an energy source since zero net CO_2_ accumulation in the atmosphere from biomass production and utilization can be achieved. The CO_2_ combustion process is compensated by the CO_2_ consumption in photosynthesis making it carbon neutral. It constitutes up to 35% of the main energy source in developing countries. However, biomass needs to be densified from the bulky mass (0.1–0.2 g/cm^3^) as pellets, briquettes, logs, or dense powders (1.2–1.4 g/cm^3^). The chemical composition and moisture content have a major effect on the final solid biofuel quality, as it influences the net heating value, ash content, and mechanical durability whereas the bulk density influences the mechanical durability, thermal characteristics, handling and storage costs (Reed et al. 1980; Shojaeiarani et al. 2019). However, the cost and energy needed for densification must be considered in deciding whether densification is practical in a given situation and location. In this regard, solar energy can be used to dry biomass using different types of dryers and flow patterns followed by densification (Prakash and Kumar 2017; Miller 2019). Solar thermal technology is rapidly gaining acceptance as an energy saving measure in agriculture application as alternative source to wind and tidal energy (Atnaw et al. 2017).

Biomass including waste biomass from agriculture residue, can be gainfully converted by using chemical, biochemical and thermochemical processes (Fig. 1). Biorefineries have been heralded partly as a promising substitution of oil refineries as well as for valorization of the entire biomass into fuel, energy and high value-added products. Its other objective is to reduce GHG and slow-down of depletion of fossil carbon. The main feedstocks for biorefinery include perennial grasses, starchy crops (wheat and maize), sugary crops (beet and cane), lignocellulosic or energy crops (switchgrass, miscanthus, willow, short rotation coppice, and poplar), lignocellulosic residues (stover and straw), oil crops (palm oil and rapeseed oil), aquatic biomass (algae, micro-organisms and seaweeds), agricultural, forest, and industrial residues (bagasse, forest thinning, stover, straws, sawdust, and paper mill waste), and consumer-generated kitchen waste (Pandey et al. 2015; Kamm et al. 2016; Lago et al. 2018; Bhaskar et al. 2020). Sustainable conversion of biopolymers such as cellulose, chitin, and chitosan-based products using (nano)-catalysis into valuable products as against fossil-fuel industry is critically analyzed (Varma 2019). Extensive literature is reported on cellulose and hemicellulose-based biofuels and chemicals as well as lignin chemistry including waste biorefinery (Bhaskar et al. 2020). Biopolymers of cellulose type, chitin and chitosan, marine algae, among other biogenic material waste can be converted advantageously to biofuels, energy products and biochemicals including niche applications in catalysis and biomedical industry (Bhaskar et al. 2020; Colmenares et al. 2017; Iravani and Varma 2020; Lucas et al. 2019).

Lignin is the second most abundant biopolymer after cellulose. Nearly 50 MMTA of lignin is produced worldwide, of which 98%–99% is incinerated to produce steam. Using kraft lignin to produce high value-added products is a great technological challenge, due to its complex structure, low reactivity, and low solubility, but can be used as lignosulfonates and dispersants, technical carbons, transportation fuels, bioplastics, and adhesives (Demuner et al. 2019). In the case of lignin, which needs to be depolymerized for conversion into useful chemicals and material, continuous flow microreactors are advocated for commercial applications (Colmenares et al. 2017). Further, interesting advancements related to the biomedical and therapeutic applications of lignin nanoparticles are discussed (Iravani and Varma 2020). Technologies for lignin conversion into activated carbon, carbon fiber production, polyurethane foam and materials, and the biological conversion of lignin with fungi, bacteria or enzymes to produce chemicals and a variety of aromatic compounds using different chemical, catalytic, thermochemical and solvolysis are presented (Fang and Smith 2016).

Integrated biorefineries use different combinations of feedstock and conversion process technologies to get a variety of products, with the main aim of producing biofuels with co-products such as chemicals (or other materials), animal feed, and heat and power (US Department of Energy 2020). Biobased fuels and chemicals must compete on cost and performance basis with petrochemicals and petrofuels. A range of products and feedstocks for chemical industry from biorefinery which are planned and under development across the world is given in Table 1. Presently, most chemical-producing biorefineries employ starch or sugar as the major feedstock using fermentation, chemical catalysis or thermochemical processes and related technologies (Fig. 1). Besides being plentiful, biomass is the only renewable resource that can currently meet society’s need for liquid fuels. Moreover, like crude oil, biomass too can be refined into a wide spectrum of products. As a consequence, the vision of biomass as a surrogate for petroleum is now a central tenet of national biomass valorization strategies the world over (United States Department of Energy (2004). Biomass Program Technical Summary 2004; Gorin 2010; Singh 2010; Star-Colibri 2011; McMillan et al. 2015).

**Table 1:** Range of products and feedstocks for chemical industry from biorefinery planned and under development across the world

1,3-Propanediol 1,4-Butanediol 3-Hydroxy propionic acid Acetic acid Acrylic acid Adipic acid Alkyl benzene Benzene Caprolactam Citric acid Epichlorohydrin Ethanol Ethyl acetate Ethylene Ethylene glycol Furan dicarboxylic acid (FDCA)	Formic acid Furfural Furfuryl alcohol Glucaric acid Glutamic acid Glycolic acid Hydroxymethyl furfural (HMF) Hydrogen Iso-butanol Iso-butene Isoprene/Farnesene Isopropanol Isosorbide Itaconic acid Lactic acid Levulinic acid and alkyl levulinates Lysine	Methane Methyl methacrylate n-Butanol n-Propanol Para-xylene PHA Phenol(s) Propylene Propylene glycol (1,2-propanediol) Sorbitol Succinic acid Toluene Vanillin Xylitol

The European Commission Directorate estimates the price per ton (in US$) for bioethanol, 1, 4-butanediol, n-butanol, succinic acid, xylitol and farnesene to be 815, 3000, 1890, 2940, 3900 and 5581, respectively (E4tech UK 2015). Some plants so built include acrylic acid, 1,3-propanediol, succinic acid, lactic acid and polylactic acid. The biobased chemicals are perceived as greener and therefore purchased despite a higher cost (Hess et al. 2016). BP also predicts a growth in the generation and use of green energy in the years ahead (British Petroleum 2019).

Diversifying the chemical industry’s resource base beyond crude oil and reconfiguring current manufacturing operations along the principles of Green Chemistry and Engineering is understandable (Anastas and Lankey 2002; American Chemical Society 2020). Unfortunately utilizing biomass as a direct substitute for crude oil makes little economic or environmental sense, and a better strategy is required. Biomass conversion, from sustainability point of view, is treated as a carbon–neutral process which will not increase global warming but not bring down the CO_2_ content already in the atmosphere unless colossal forestation programs are undertaken but what is desirable is the carbon negative processes which will be dealt with later. Putting an overemphasis on biofuels derived from crops, such as corn, sugarcane, soybeans, wood and agricultural residues for use as renewable energy sources have been questioned from the view point of environmental problems, food versus feed supply, and serious destruction of vital soil resources (Pimentel, 2008). A theoretical basis with exhaustive evidence for the case against large scale biofuel production using agricultural crops is advocated and its devastating impact on biodiversity is pointed out because of additional loss of habitat for agriculture and accompanying rural development due to the additional pressure on traditional farming (Giampietro and Mayumi 2009). The concept of so-called CO_2_ refinery will be relevant using hydrogen as a reactant for carbon derived economy which will be discussed later.

**Fig.3:**
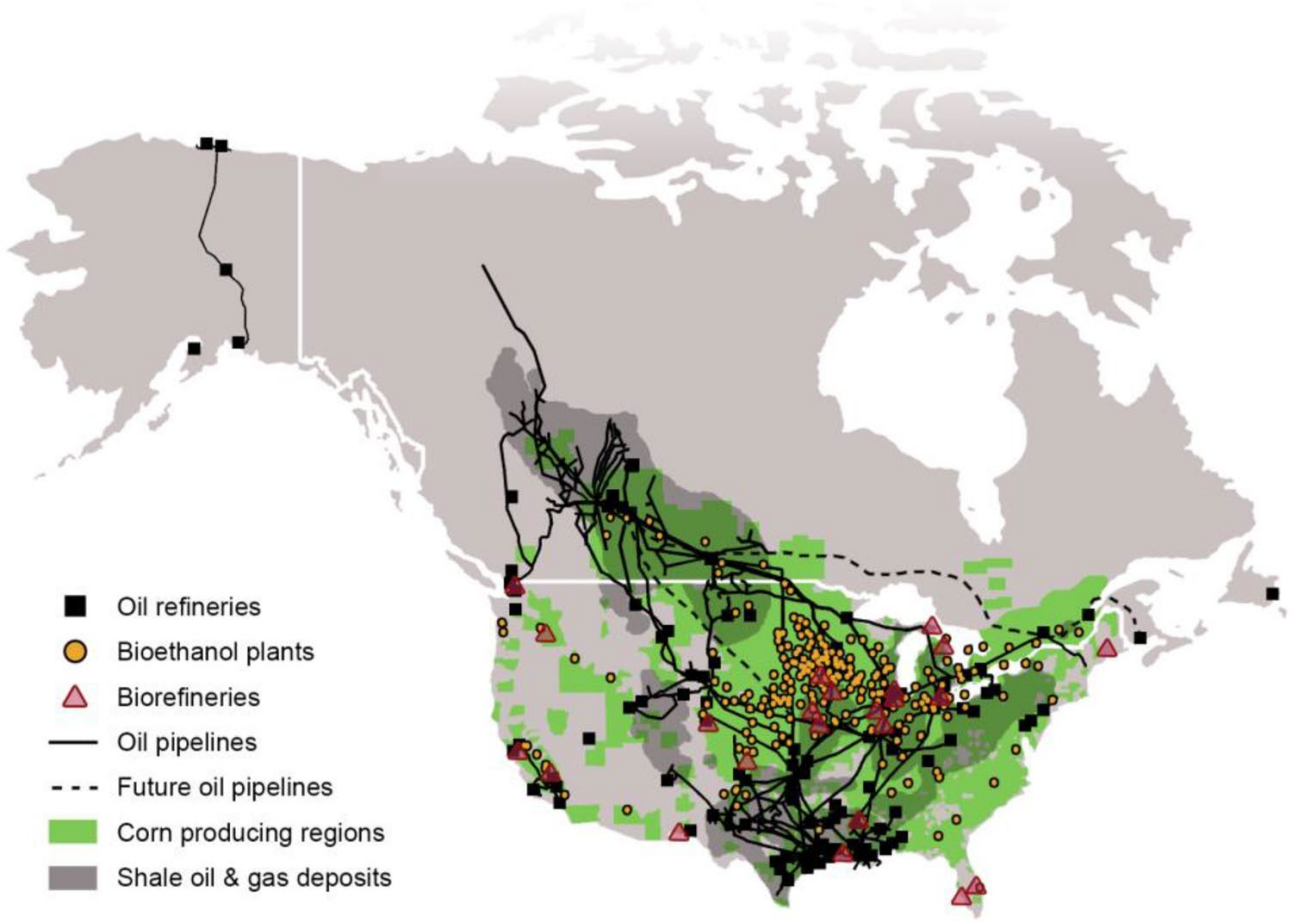
The network of transportation pipelines in North America bears little overlap with its biomass producing belts, which raises transportation costs and lowers the ‘crack spread’ for biorefining

What are the problems and technological challenges associated with voluminous quantities biomass spread on huge land mass? At the outset, biomass is incompatible with the current refining infrastructure. Unlike crude oil, biomass is susceptible to spoilage, it has humongous volume and its caloric content too is significantly lower. Collection, densification, and transportation of biomass is very expensive. As a consequence, biorefineries are geographically constrained to be located close to sources of biomass. Across the world generally and in North America particularly, since the existing liquid transportation infrastructure overlaps minimally with biomass production zones (Fig. 3), biorefined products incur exorbitantly high transportation costs, which thins the ‘crack spread’ for biorefining. This implies that biorefineries are not only sensitive to fluctuations in the costs of biomass and biorefined products, but also require the price of oil to be sufficiently high just to break even. As a comparison, if the price of a barrel of oil falls below $70, using shale gas as a feedstock for manufacturing fuels and chemicals is no longer profitable (Dimick 2014). The prices was less than $43 in July–August 2020 but will again rise in next 2–3 years once the Corona virus effect is subsided. This hampers the use of biomass as a feedstock unless it is aimed at converting waste biomass as a pollution abatement cost and not a profitable business. This is precisely the case with second generation (2G) ethanol manufacturing processes; that industry gets huge subsidy. When one considers that biorefining is neither as mature nor efficient as valorizing shale gas, it implies that the minimum price of a barrel of oil in order for biorefineries to break even should be much higher. It is apparent that biorefineries are at a significant competitive disadvantage in the new era of cheap oil as of now and the imminent shale gas revolution (PwC 2013). Although the oil industry and global economy will look very different once the world has recovered from the COVID-19 crisis (a topic of discussion that is beyond the scope of this article, a superficial assessment of global economy suggests that the competitive disadvantages of biorefining are only going to worsen in a post-COVID-19 world). Additionally, the geographical constraints of biorefineries also present a formidable challenge to achieving economies of scale (Richard 2010; Sims et al. 2010), which has been one of the central drivers of innovation in the petrochemical industry.

The incompatibility of biomass with the refining infrastructure also extends to its chemical composition. The high oxygen content of biomass and its distinct chemical bonding patterns make it recalcitrant to most of the thermochemical transformations that are commonly employed in oil refining. As a consequence, fractionating biomass in a manner akin to crude oil necessitates use of specially tailored catalysts, solvents for dissolution, and novel chemistries (Rinaldi 2014), neither of which have been developed to a satisfactory standard, as well as significant quantities of reagents, which greatly lowers the atom economy of biorefining. Incidentally, petroleum refining generates less than 0.1 kg of waste for every 100 kg of products (Sheldon 2007), making it one of the most efficient industrial operations in the world today. The sustainability issues with reference to chemical technologies and waste minimization which are applicable to refineries have been investigated previously (Sikdar 2007) and a projection of the green chemical industry by 2100 is presented. A stronger argument for using biomass as a feedstock could be made if it were utilized for manufacturing products that are distinct from petroleum-derived products and if suitable thermochemical conversion technologies existed (Nogueira et al. 2013; Sheldon 2016; Lane 2016). This is clearly not the case for biorefining. In fact, the curious decision to employ conventional thermochemical processes to refine biomass into products that are otherwise derived from petroleum makes us question the rationale for using biomass in the first place.

It is not the refining of crude oil but the combustion of its products that has a deleterious effect on the environment. It is, therefore, inappropriate to suggest that replacing crude oil with biomass without significantly altering the product spectrum will mitigate climate change. If anything, using suboptimal conversion technologies to produce the same products as before will only worsen the problem. The only plausible justification to move away from crude oil is that of resource scarcity and here too, the argument is a weak one. For a brief period, debates about peak oil raised prospects of a post-petroleum age. However, as extraction technologies have advanced over the course of the past decade, well-productivities too have risen, which now means that global oil reserves are actually expected to double within the next 4 decades (Reuters 2015). This fact, along with the rapid emergence of shale gas as a feedstock for the production of energy and chemicals, has meant that fears about hydrocarbon scarcity have entirely dissipated. Nonetheless the production of chemicals from biomass by using smart technologies should be a long-term goal before the time the world runs out of crude oil.

## A path forward: convert, not refine

**Fig.4:**
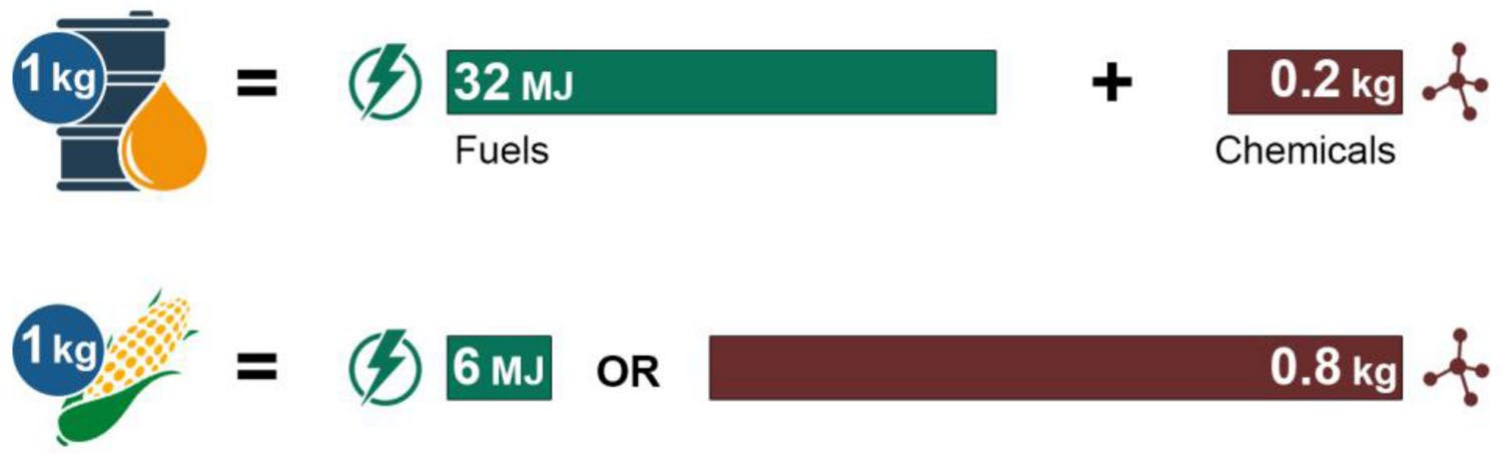
Energy and mass balances on crude oil and biomass reveal that the latter is better suited for use as a feedstock for chemical manufacturing

Although we are opposed to refining biomass solely for biofuels as a future strategy, for instance by 2030, we believe that, like crude oil and shale gas, it will play an important role in shaping the future of the chemical industry as synthetic catalytic chemistry matures and synthetic biology emerges. However, unlike biorefining, wherein it is treated as a renewable surrogate for crude oil, we instead view biomass as a complement to crude oil and shale gas and advocate its thermochemical, chemical or biological conversion to chemicals and materials, not fuels. Refining principally involves multitudinous exchanges of C–C σ-bonds and C–H bonds within the feedstock for more reactive C–C π-bonds. These transformations break apart the molecular structure of the feedstock, and the π-bonds subsequently react with one another to generate a plethora of products that are distinguished by their volatilities. Conversion, on the other hand, involves significantly fewer and more selective exchanges of less reactive bonds for more reactive ones, which then generate a vastly smaller number of products, but at higher yields and high selectivities. Our recommendation is based on mass and energy balances on crude oil and biomass (Fig. 4), as well as an appreciation of the rate and scale of oil refining. Presently, crude oil is society’s dominant source of chemicals and only source of liquid fuels. Each kilogram of crude oil yields roughly 0.2 kg of chemicals and 0.8 kg of liquid fuel products, the latter of which have a cumulative caloric content of 32 MJ (Rødsrud et al. 2012; Vesbrog and Jaramillo 2012). In comparison, if one assumes that biomass can be converted to ethanol at theoretical yields, a kilogram of biomass yields no higher than 6 MJ of energy. This estimate is based on the highest yields reported for pre-treatment and saccharification of corn and the fermentation of sugars produced thereof, which, it must be noted, ranks amongst the most efficient modes of converting biomass to liquid fuels. The true figure for energy derived from a kilogram of biomass is surely lower.

**Fig.5:**
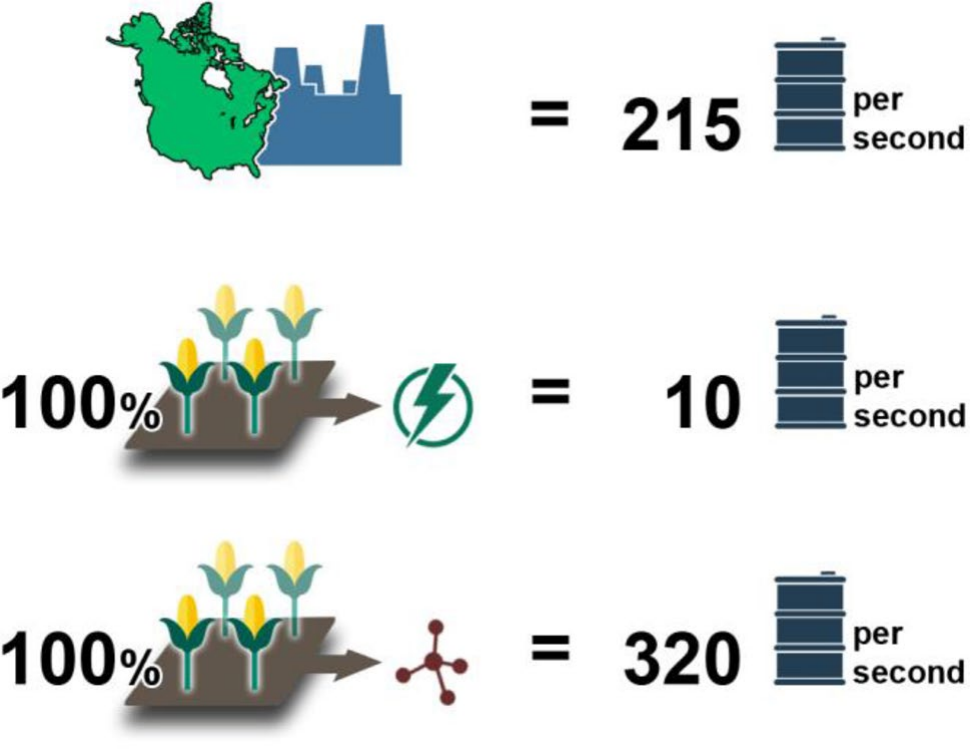
The difference between the fuel and chemical production capacities for biomass, when scaled to refinery output, is even wider, thereby corroborating our recommendation that biomass should be used to manufacture chemicals and not fuels

Alternatively, if biomass is exclusively diverted to the production of chemicals, it can yield approximately 0.8 kg of products on a per kg basis. The difference between the conversion capacities of biomass to fuels and chemicals, respectively, becomes more emphatic when the conversions are scaled to refinery output of North America, which is approximately 215 bps (Fig. 5). If all the corn produced in North America were converted to ethanol, the total energy derived from this conversion is equivalent to a mere 10 bps.

Even if one sets aside the food-versus-fuel debate (Tenenbaum 2008; Kline et al. 2016) and assumes that the production and combustion of biofuels is carbon neutral—a fairly contentious claim (Fargione et al. 2008), converting biomass to biofuels has a maximum oil replacement capacity of less than 5%. On the other hand, if the same amount of corn were diverted to chemical production, roughly 320 bps of crude oil usage can be replaced**.** Moreover, employing biomass as a feedstock for chemical manufacturing also offers a wider crack spread—or more appropriately, higher valorization spread. For instance, lignin comprising 30% oxygen costs roughly 5 cents per kilogram in fuel equivalent value. However, while petrofuels refined from this feedstock cost no more than $1 per kilogram, speciality aromatic chemicals derived by the catalytic depolymerization of lignin fetch, on average, as much as $15 per kg. In fact, some chemicals such as vanillin can command as high as $100 per kg. Besides, refining biomass into petrofuels consumes a significant amount of hydrogen, which greatly lowers the atom economy of the process.

## Biomass to biofuels: a broken concept

**Fig.6:**
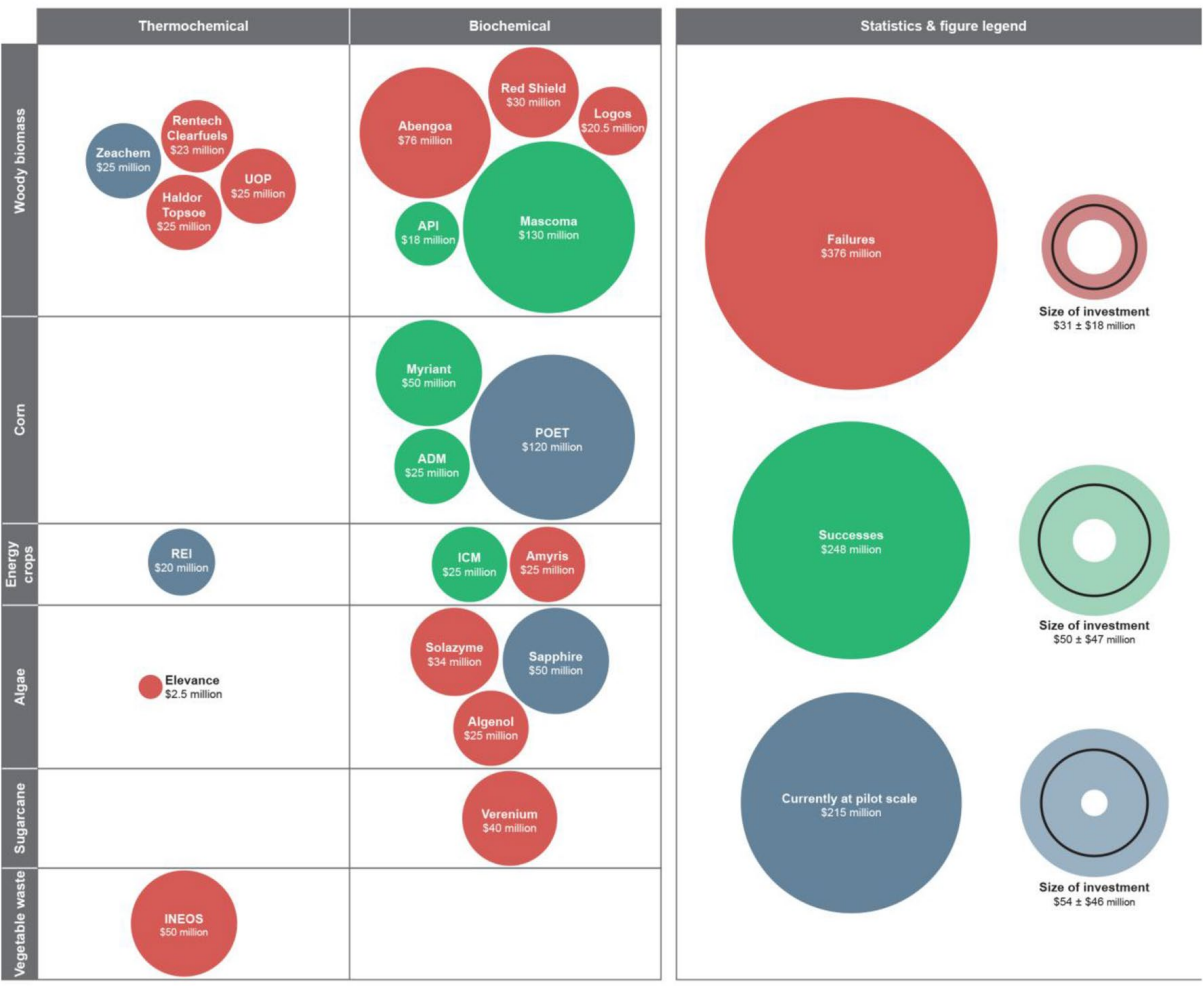
Investments by the US Department of Energy on biofuels companies have, more often than not, ended in losses. Detailed information about the companies listed in the figure is available in the Supplementary Information that accompanies this article

Based on the critical analysis of published data from various sources, our recommendation against using biomass as a feedstock for low cost high volume liquid fuels or fuel additives runs contrary to the strategy of the US Department of Energy, which has invested aggressively in recent years to establish integrated biorefineries across the United States that are capable of converting locally available biomass to cellulosic ethanol and bio-power [US Department of Energy (2013), DOE/EE-0912 (2013)]. This policy has been replicated in other countries like Brazil and India. Scores of articles have been written on fuel additives based on bioresources from the environmental perspective. In fact, it has been mandated in several countries to mix gasoline and diesel with bioethanol as a strategy to combat pollution as well as to supplement farmers income by using agricultural residue to have 2G ethanol manufacture. It also reflects their commitment to the Paris Agreement on Climate Change (UN Framework Convention on Climate Change 2015). Does it make a commercial sense or are there better options for pollution free transport sector such as electricity or hydrogen, fuel cells and the like and use biomass for production of chemicals and chemical feedstocks? However, the litany of failures of biofuel manufacturers in the United States (Fig. 6) supports the foregoing conclusion on biofuels. It is clear that the favorability in conversion efficiencies and valorization potentials for converting biomass to low-volume, high-value chemicals over high-volume, low-value fuels and fuel additives also extends to the profitability of the manufacturing processes and their immunity to market fluctuations.

Biofuel manufacturers require considerable investments—not to mention favorable regulations and geographical proximity to distribution pipelines—if they are to succeed, and here too, it is imperative for such ventures to diversify into chemical manufacturing (**Supplementary Information**). Nevertheless, one speculates about the long-term prospects of the surviving biofuels ventures in the United States, especially since the atom, energy and cost disadvantage of converting biomass to fuels is only going to widen in the new era of cheap and abundant crude and shale gas. Further electric transportation has begun to make significant inroads into the automobile market. Countries such as the UK, France, and India have long range policies to ban the combustion engine in new vehicles and automobile companies such as Volvo, Telsa, and Nikola, for instance, have signaled new cars will be electrified. As such the return on a $300 to $500 million biorefinery creates additional risk to the investors with a minimum return within 7 years, calls into considerable question where transportation technology will be by 2025.

## The new bioeconomy

**Fig.7:**
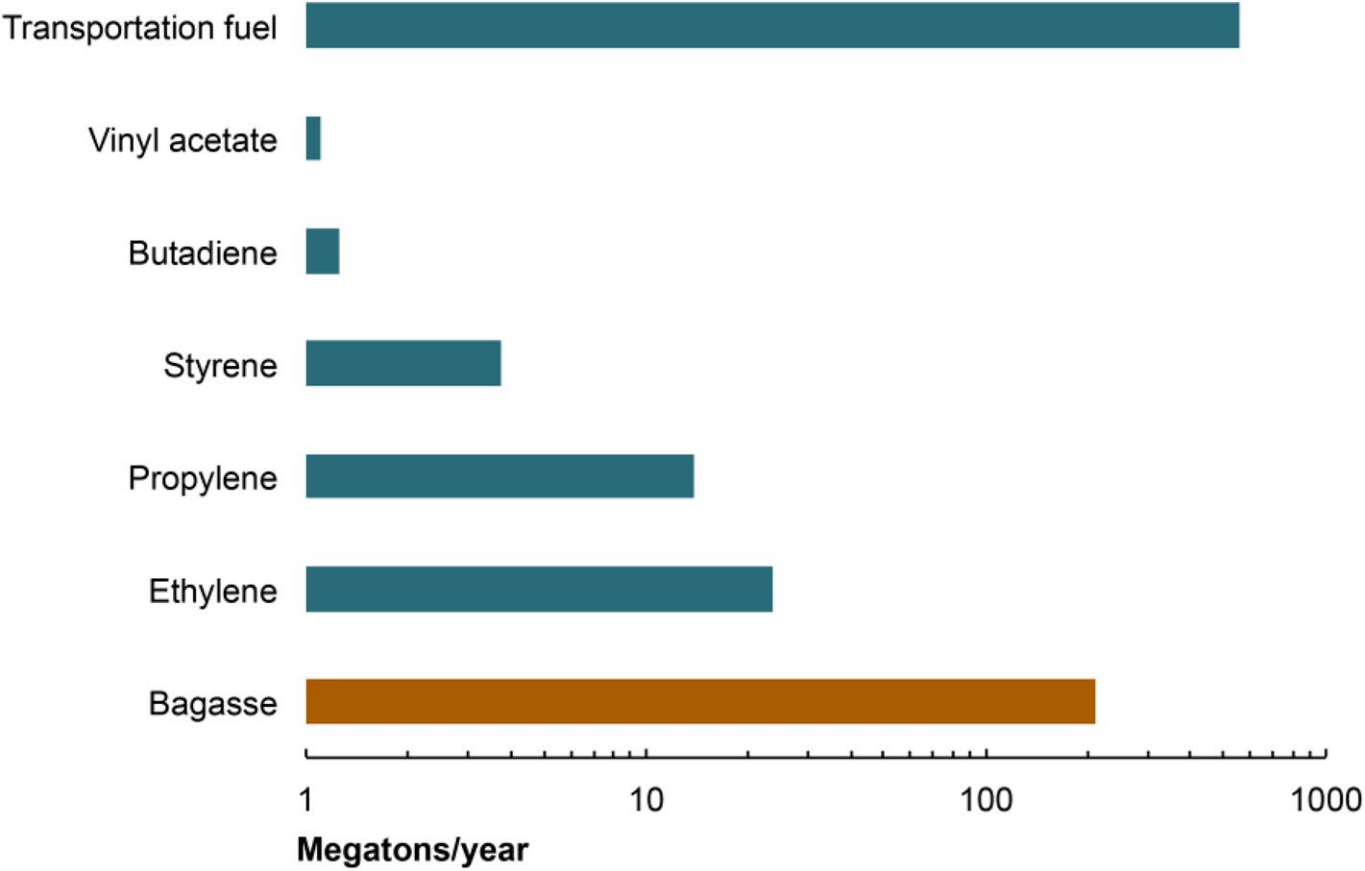
Production of selected petroleum-derived products in North America. Transportation fuels comprise nearly three quarters of the refinery output in North America, whereas ethylene and propylene account for the largest share of petrochemicals. This product distribution is similar across the world. As a comparison, North American bagasse production is estimated to be roughly 210 megatons each year

Nearly a quarter of the crude oil that is consumed in North America is employed for non-transportation uses such as petrochemical production, of which ethylene comprises the dominant fraction (Fig. 7) (Tuck et al. 2012). This distribution is roughly similar across the world. However, since petrochemicals command higher prices than fuels, it is the demand for the former that is a stronger driver of refinery output.

**Fig.8:**
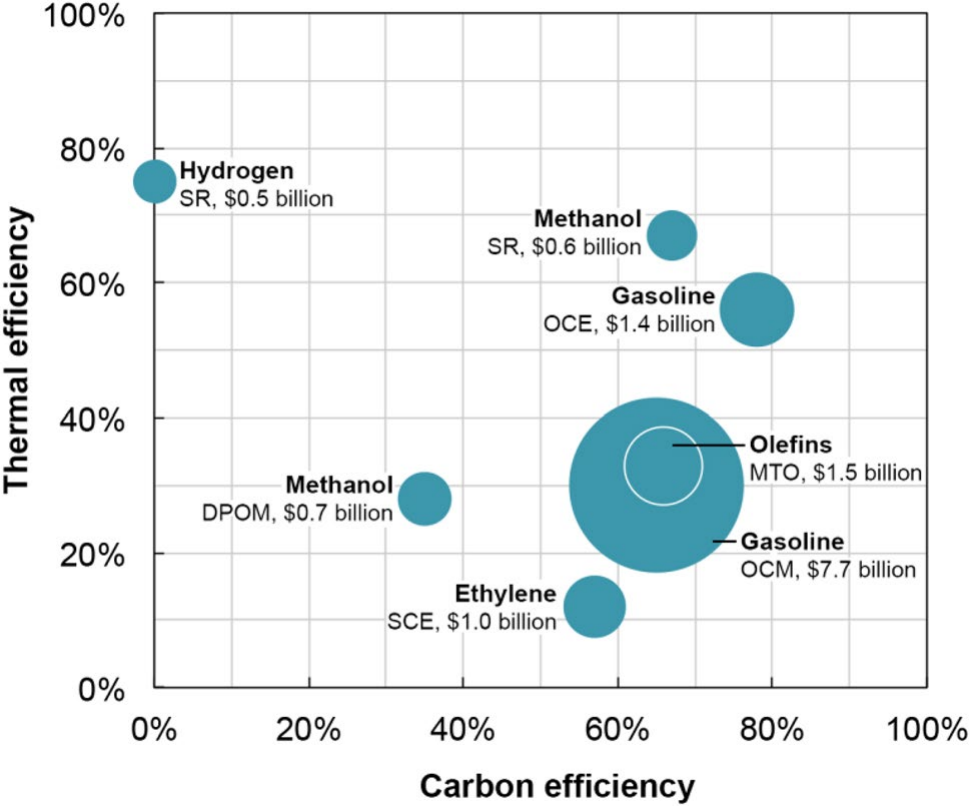
Methane conversion processes such the MTO process and OCM exhibit higher thermal and carbon efficiencies compared to SCE, the dominant process for ethylene production. The bubbles in the figure represent current capital costs for plants with a processing capacity of 12,000 bpd

As a consequence, replacing petrochemicals with chemicals derived from shale gas and biomass could substantially deflate oil consumption without significantly affecting the dynamics of the fuel market. In fact, eliminating petrochemical production from oil refineries achieves even higher atom and energy economies through improved process integration (Allen 2007). As a corollary, utilizing shale gas as a feedstock for the manufacture of commodity chemicals such as ethylene greatly improves its valorization spread. Presently, as much as 80% of the shale gas that is fracked in North America is converted to LNG, which is almost exclusively used as a source of fuel, heat or electricity (Stangland 2014). Instead, converting the methane and ethane in shale gas into commodity chemicals is a significantly superior strategy for resources monetization. In the United States, for example, steam cracking of ethane (SCE) is employed to produce nearly 24 kilotons of ethylene each year, and the increasing availability of domestic shale oil and gas has proved to be a shot in the arm for the US chemical industry. The American Chemical Council estimates that as many as 148 projects totaling over $100 billion dollars in new capital investments will be floated over the next 10 years to take advantage of the shale gas boom. Methane can also be used as a feedstock for the manufacture of hydrogen (for ammonia production), syngas, methanol, gasoline and electricity (Wolf 1992; Hackworth et al. 1995; Lange and Tijm 1996; Rostrup-Nielsen et al. 2000; Lange 2001; Rostrup-Nielsen 2002; de Klerk 2015); and this proposition is corroborated by a detailed assessment of the carbon and thermal efficiencies and capital costs for a suite of methane-to-chemicals processes (Fig. 8).

In fact, methane-to-olefins (MTO) conversion processes and oxidative coupling of methane (OCM) are more thermodynamically and carbon efficient than steam cracking of ethane (SCE). The thermal efficiency of a process denotes the extent to which energy added as heat is converted to work. Carbon efficiency, on the other hand, is defined as the ratio of carbon that is present in all significant products to the carbon present in all reactants. The capital costs listed Fig. 8 are estimates, which are adjusted for inflation, for plants that have a processing capacity of 12,000 bpd and utilize the best available technologies in 1995 (Fox 1993; Lange and Tijm 1996; Basye and Swaminathan 1997; Lange 1997; de Pontes et al. 1997). Additionally, the estimates exclude costs for product separation, upgrading and transportation. Nevertheless, since MTO processes are at a much earlier stage of their technological life cycle compared with SCE, additional investments and R&D will be required for the maturation of MTO conversion technologies. Additionally, although the lower feedstock cost for methane sufficiently compensates for the high capital costs associated with methanol to olefin (MTO) and oxidative coupling of methane (OCM), at current crude oil prices, it is more prudent to focus on MTO conversion over producing liquid transportation fuels via oxidative coupling of methane (OCM) (Lange and Tijm 1996; Lange 2001). This conclusion resonates with our proposal to utilize crude oil, biomass and shale gas as a source of fuels, value-added chemicals and commodity chemicals, respectively.

**Fig.9:**
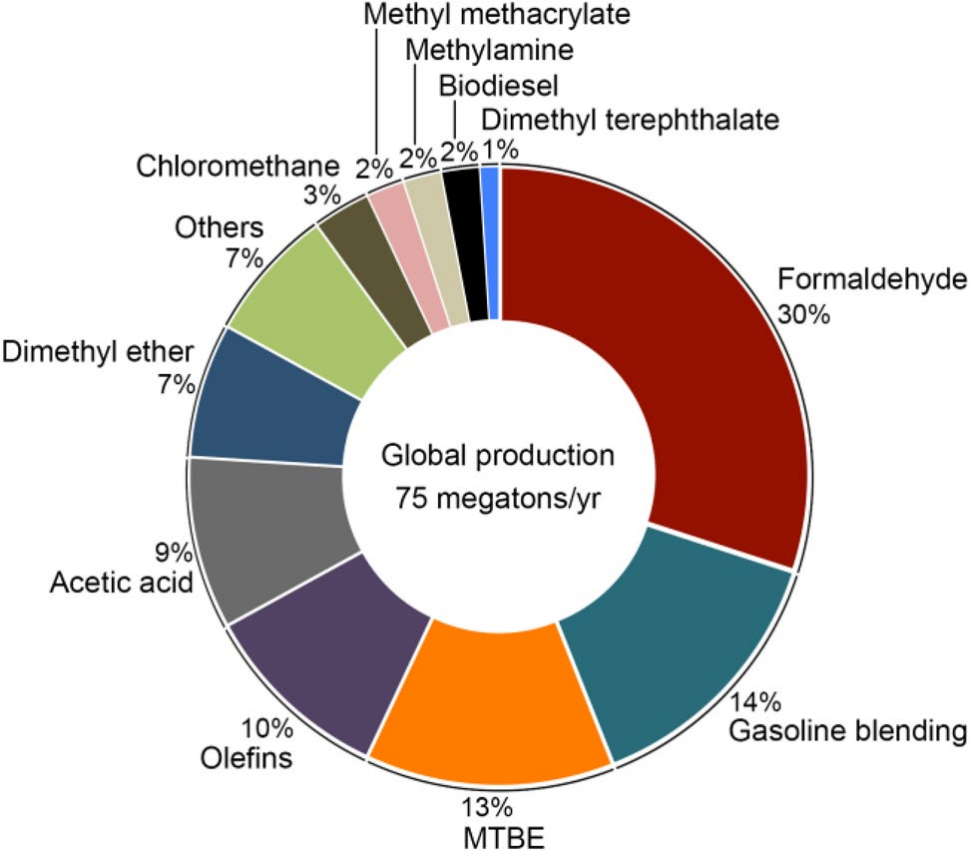
Methanol is a versatile feedstock for the production of fuels and chemicals, although we advocate reserving it for chemical manufacturing

On the other hand, methanol can be synthesized from methane either through steam reforming (SR) or direct partial oxidation (DPOM) or dry reforming (DR) with carbon dioxide. Of these, SR and DPOM are comparably economical, although SR is at a rather advanced stage of technological maturation and expectedly has greater thermodynamic and carbon efficiencies compared to DPOM. Nevertheless, the economical use of methane as feedstock for the production of methanol lays strong foundations for the future methanol economy (Fig. 9) (Olah 2005).

**Fig.10:**
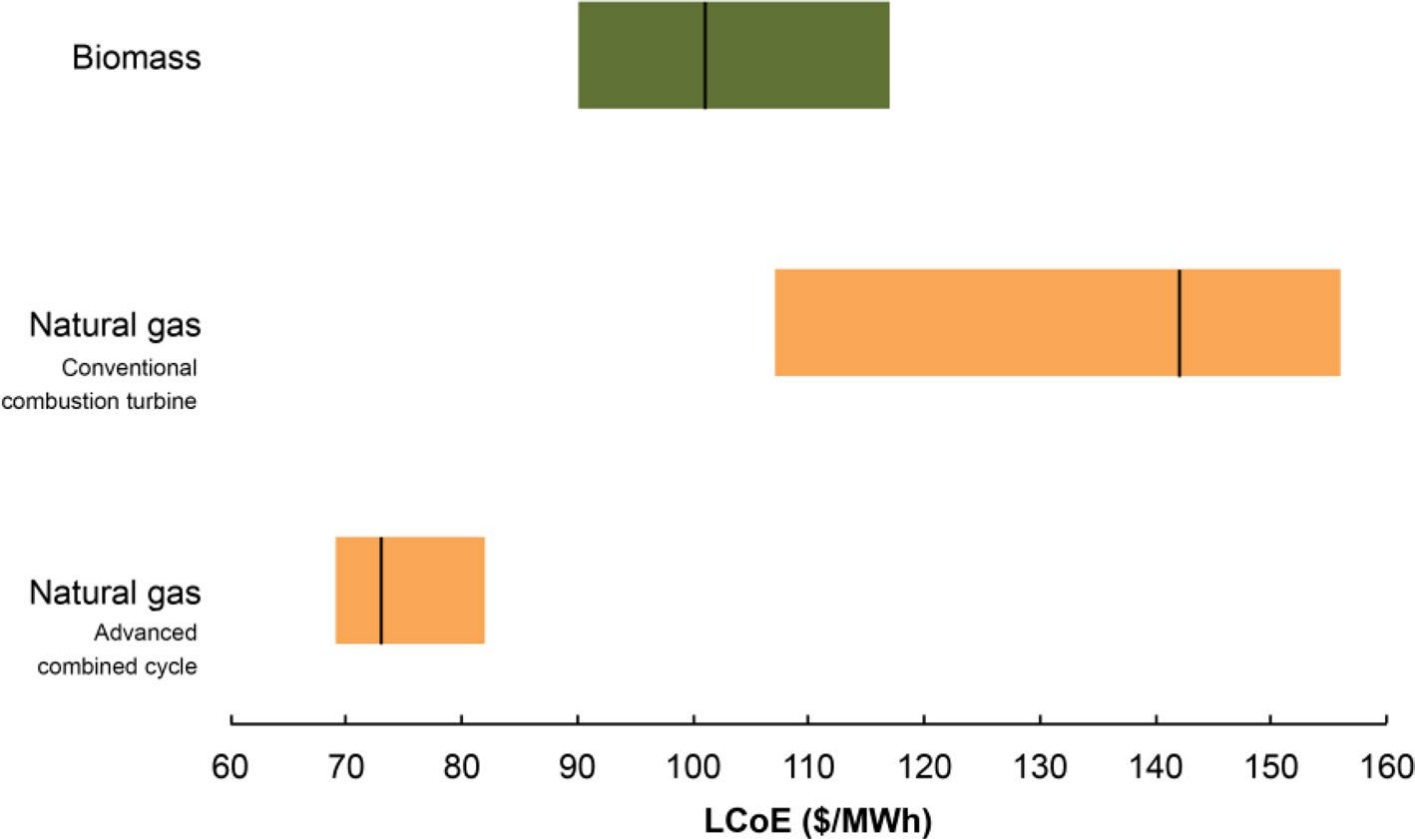
The levelized cost of electricity (LCoE) generated by combined cycle power plants fueled by natural gas is substantially lower than power plants that either consume biomass or employ gas turbines. The extremities of each bar represent the minimum and maximum costs, whereas the vertical black line that intersects each bar denotes the average levelized cost

Finally, shale gas can also be utilized for generating electricity, and the levelized cost of electricity generated by advanced combined cycle power plants fueled by natural gas is quite competitive (Fig. 10) (US Energy Information Administration 2020).

**Fig.11:**
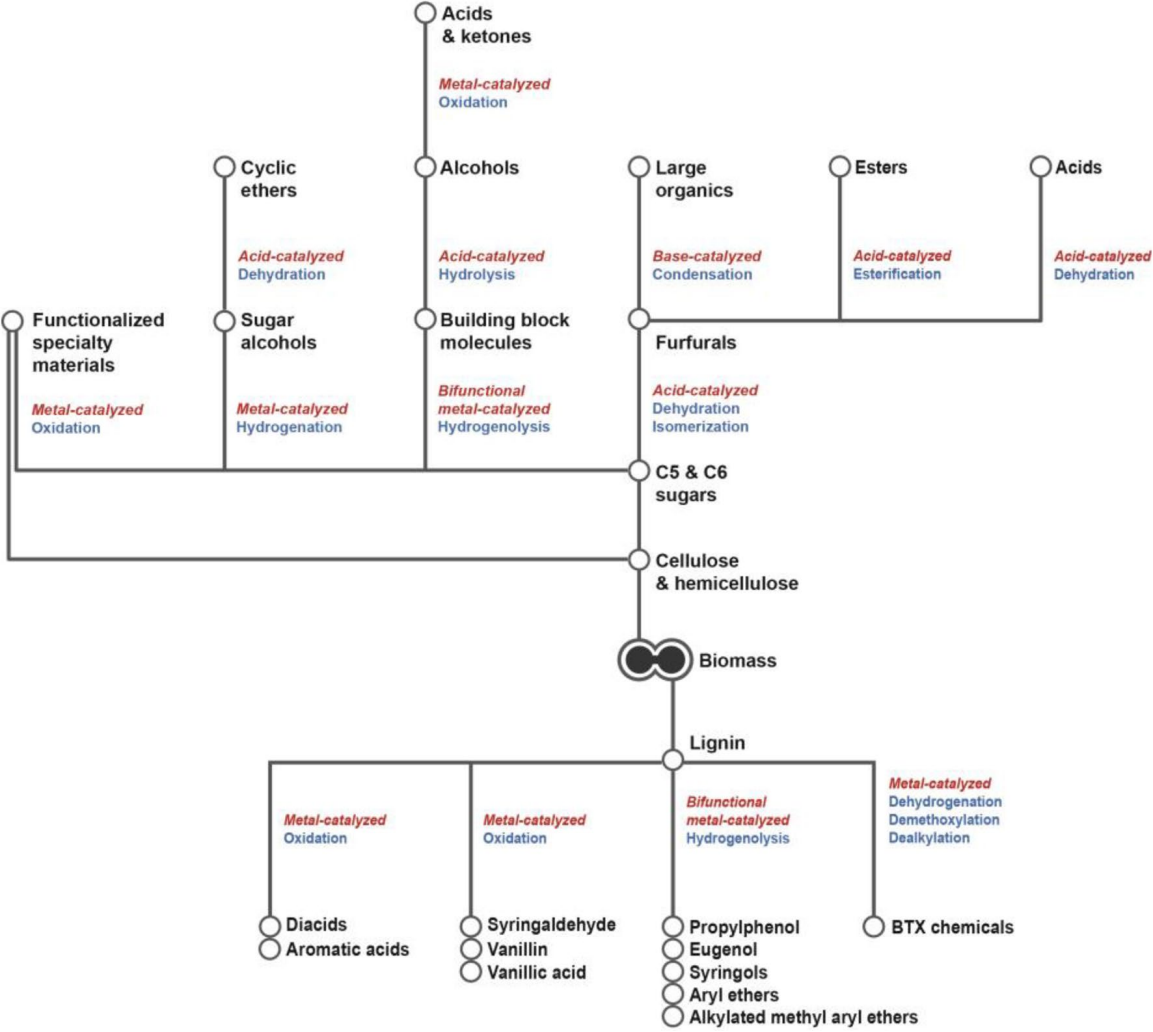
A rich catalog of catalytic processes are available for producing value-added chemicals from biomass

Numerous catalytic (Fig. 11) and biocatalytic processes that convert lignocellulosic biomass into value-added chemicals have been developed in recent years (Serrano-Ruiz et al. 2010; Bornscheuer et al. 2012; Gallezot 2012; Sainsbury et al. 2013; Besson et al. 2014; Sheldon 2014, 2016; Strachan et al. 2014; Hess et al. 2016; Upton and Kasko 2016). While the number of biocatalytic processes that directly convert lignocellulose into value-added chemicals is still small, the increasing number of examples involving the synergistic application of metagenomics and enzyme & metabolic engineering for lignocellulose bioconversion augurs well for the future development of improved bioprocesses for the selective synthesis of tailor-made chemicals (Strachan et al. 2014; Pawar et al. 2016; Hess et al. 2016).

## Hydrogen economy and CO_2_ refinery

**Fig.12:**
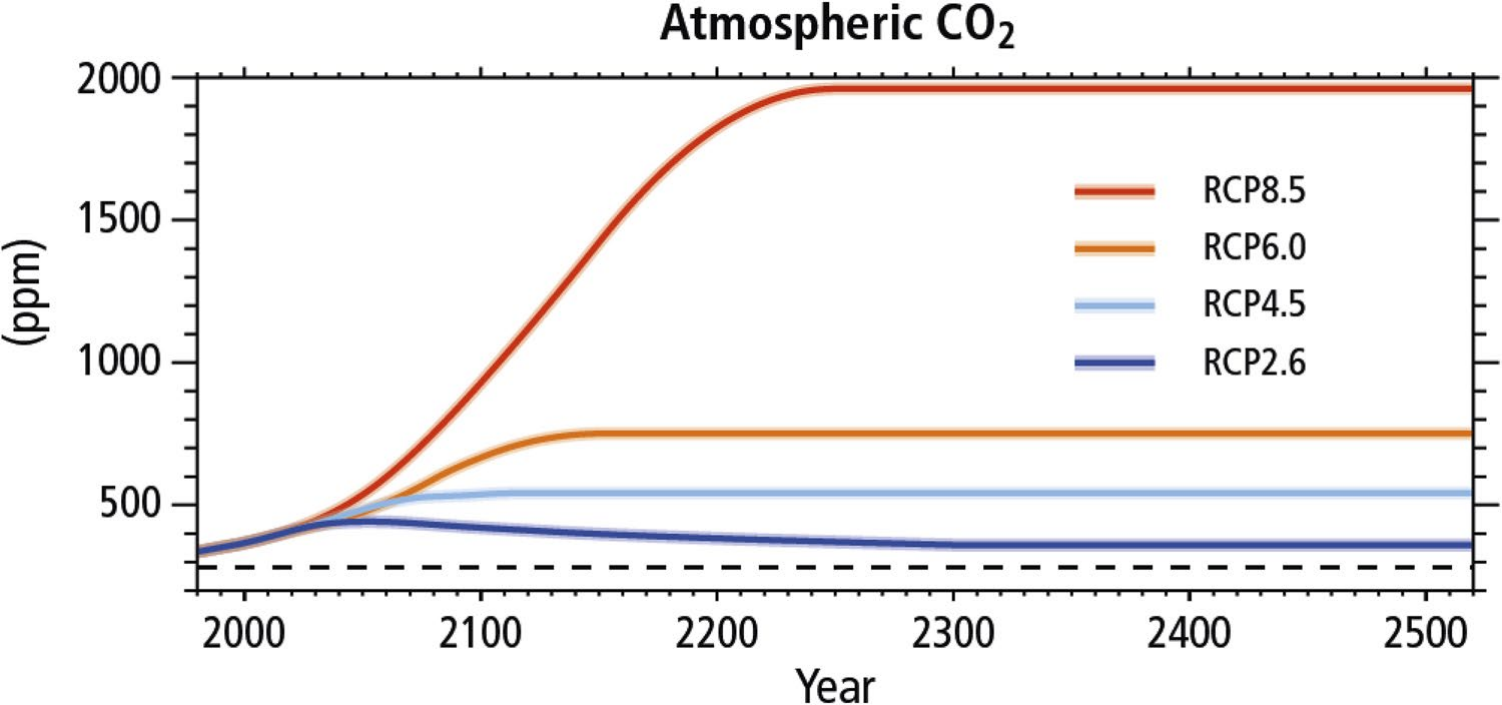
Impact of CO_2_ emissions in atmosphere if no technological intervention is done ( Source: IPCC Intergovernmental Panel on Climate Change show projected concentrations of CO_2_)

**Fig.13:**
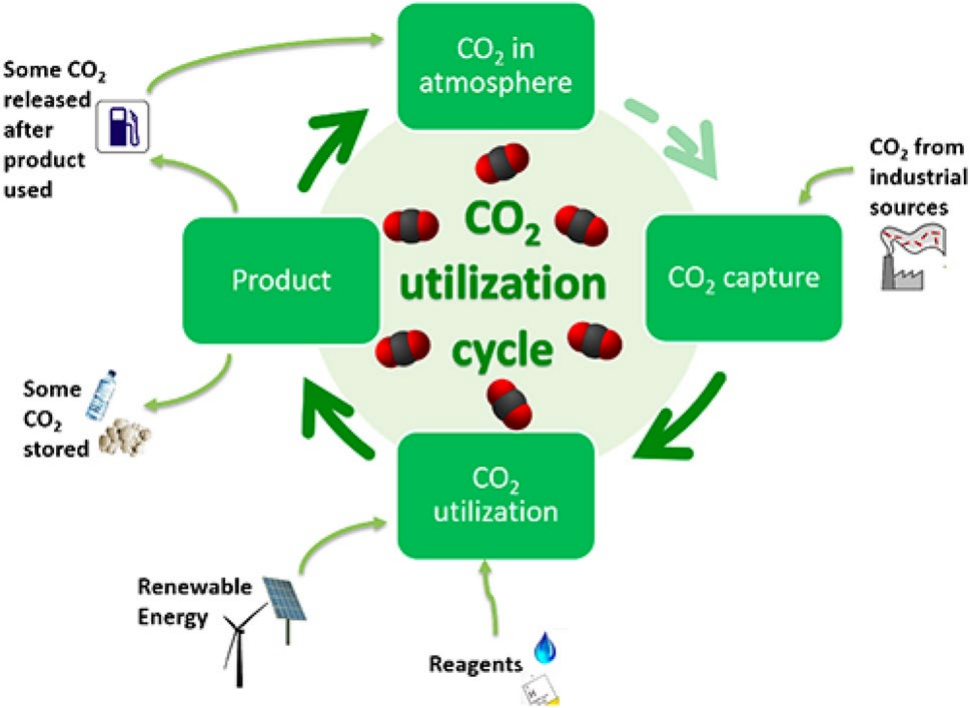
The industrial carbon cycle

Whether the feedstock is based on crude oil or biomass, the ultimate fate of the carbon is carbon dioxide in the atmosphere which is responsible for global warming (Fig. 12). It is thus imperative to convert CO_2_ into valuable chemicals, fuels and materials (Fig. 13). The so-called carbon neutral economy, based on biomass, should be outdated if the temperature of the globe has to be contained within 1.5–2 °C by the end of twenty-first century. To bring down the CO_2_ content in atmosphere from the current ~ 410 ppm to a level below industrialization would require massive efforts to convert CO_2_ into chemicals and materials. The so-called CO_2_ refinery necessarily uses hydrogen to make syngas and other chemicals.

The use of hydrogen among other renewable energy sources is hotly pursued in the hydrogen economy for energy and chemicals (Nazir et al. 2020). In other words, hydrogen will play a critical role in not only achieving the objective of converting CO_2_ into fuels and chemicals such as methanol, dimethyl ether, formic acid, hydrocarbons and polymers but also transforming (waste) biomass into fuels and chemicals (Mondal and Yadav 2019a, b). The conversion of methanol into a variety of chemicals was already discussed.

**Fig.14:**
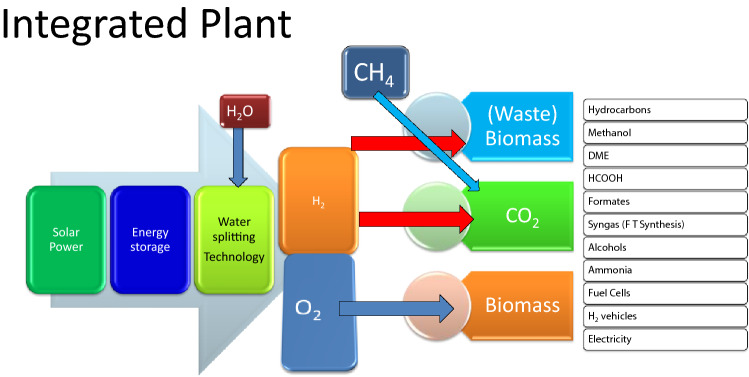
Hydrogen valorization pathways

Formic acid is a store house of hydrogen whereas DME is an excellent substitute for LPG and diesel. Hydrogen will lead to carbon-negative systems much before when oil is depleted totally which will also be in tune with the Paris Agreement. Hydrogen economy is a separate subject but H_2_ can be produced from biomass, bio-based alcohols like methanol, ethanol, n-butanol and ethylene glycol, methane and water splitting using different techniques. Figure 14 shows the integrated plant for hydrogen production from water splitting and its use in controlling environmental pollution and climate change as well as production of many chemicals. Helm (2102) had argued for a new, pragmatic rethinking of energy policy—from transitioning from coal to gas and eventually to electrification of transport, to carbon pricing and a focus on new technologies. Our analysis shows why and how the energy and material policies should take into account carbon for chemicals and materials with non-carbon renewable sources of energy.

## Conclusions

The over-use of fossil products has wreaked considerable damage on the environment. These concerns have re-invigorated the quest for alternatives to crude oil and were the basis for the establishment of Mission Innovation, a global initiative comprising 24 nations, including the United States, China and India. Mission Innovation aims to address climate change, make clean energy more affordable and strengthen the green economy by accelerating public and private clean energy innovation, and its most recent ministerial meeting took place in Vancouver, Canada in 2019. Experts at the helm of Mission Innovation strongly believe that the best strategy to decarbonize the global economy is to use biomass as a surrogate for oil, and the organization has invested upwards of $10 billion to bring this vision to fruition. Even the International Energy Agency supports and reinforces this idea at every opportunity. Herein, we have argued that using biomass in the manner proposed by Mission Innovation will not address any of its goals. Biomass is chemically and geographically incompatible with the existing refining and pipeline infrastructure, and achieving economies of scale necessitates integration of biomass production with the refining infrastructure, which greatly slashes the margins of refiners without mitigating any of their risks. Moreover, the environmental footprint of thermochemical refinement of biomass is not any less or more than refining crude oil using the same operations. The practice of converting biomass into liquid fuels or fuel additives should also be discontinued. Instead, we sincerely hope that governments re-configure their biomass valorization strategies and prioritize the development and technological maturation of large-scale MTO processes, among others, and catalytic and biocatalytic processes for converting biomass into value-added chemicals and functional materials. This strategy delivers a higher valorization spread for shale gas and biomass, greatly improves the atom and energy economy of oil refining, and depreciates oil use, which, in turn, mitigates carbon emissions significantly. Hydrogen is going to play an important role in the clean energy sector as well as CO_2_ mitigation by converting into a spectrum of chemicals and materials and also in conversion of waste biomass. Using ambient carbon dioxide for beneficial purposes requires an in-depth thermodynamic analysis, which is mostly unfavorable from entropy and free energy consideration. Carbon dioxide conversion to syngas, dimethyl ether, methanol and other chemicals has been studied widely using different catalysts. The commercial aspects are challenging. Carbon dioxide methanation to syngas is thermodynamically favorable. A number of hydrocarbons can also be produced.

Though power was not a focus of this paper, it is inseparable from a discussion of the mix of fuels that can be used. Shale gas is a case in point. This as a source of power has been acknowledged, but its global potential (currently limited to the US) as a replacement of coal and biomass has not been stressed. This is important in the face of the inevitability of electric cars and trucks soon.

## Electronic supplementary material

Below is the link to the electronic supplementary material.

Supplementary file1 (PDF 77 kb)

## Abbreviations

$: United States dollar
Bbl: Barrels or barrel equivalents of oil
Bpd: Barrels or barrel equivalents per day
Bps: Barrels or barrel equivalents per second
DPOM: Direct partial oxidation of methane
DR: Dry reforming
LCoE: Levelized cost of electricity
LNG: Liquified natural gas
MMBD: Million barrels or barrel equivalents per day
MJ: Megajoules
MTO: Methane-to-olefins
OCM: Oxidative coupling of methane
R&D: Research and development
SCE: Steam cracking of ethane
SR: Steam reforming
