# Supplementary material for: The production of fuels and chemicals in the new world: critical analysis of the choice between crude oil and biomass vis-à-vis sustainability and the environment
Source: Clean Technol Environ Policy. 2020 Sep 21;22(9):1757–74. doi: 10.1007/s10098-020-01945-5 (PMC7505498; doi:10.1007/s10098-020-01945-5)
Supplement: Supplementary file 1 — Supplementary file1 (PDF 77 kb) [file 10098_2020_1945_MOESM1_ESM.pdf]

Supplementary Table S1: USA bio-business landscape

**Company name**	**Location**	**Feedstock**	**Technology**	**Primary/ major products**	**Capacity (gal/yr)**	**Status**	**Comments**	**Ref.**
Red Shield Acquisition, LLC	Old Town, ME	Woody biomass	Biochemical	Ethanol, fuel oil	555,000	**Bankrupt**	Woody biomass to energy	[1,2]
Renewable Energy Institute	Toledo, OH	Agricultural residues, algae, woody biomass, energy crops	Thermochemical-gasification	Renewable hydrocarbons	6,25,000	**Pilot scale**	Integrated Biorefinery Platform	[3]
American Process Inc.	Alpena, MI	Woody biomass, hardwood derived hydrolysate	Biochemical	Ethanol	8,94,000 + 696000 potassiu m acetate	**Operational with profits**	The biorefinery supports supply chain alliances with downstream end-users of bio-based sugars, lignin, and cellulose for conversion into renewable, bio-based chemicals, fuels, and materials.	[4]

Mascoma Corporation	Kinross, MI	Hardwood, pulpwood	Biochemical	Ethanol, heat and electricity from lignin and bark	20,000,000	**Operational with profits**	Many companies involved in project: Valero Energy Corportation Michigan Economic Development Corporation (MEDC) Oak Ridge National Laboratory (ORNL) Purdue University New York State Energy Research and Development Authority (NYSERDA) New York State Power Authority (NYPA) J.M. Longyear, LLC Frontier Renewable Resources, LLC.	5
Elevance Renewable Sciences	Bolingbrook, IL	Algae oil, plant and animal oil	Chemical treatment	Initially hydrocarbons, later pivoted to speciality chemicals such as long-chain methyl esters and diacids	N/A	**Shutdown in 2010 due to inability to put together private funding,** **restarted with new product range renewicals ™**		6,7

Haldor Topsoe Inc.	Desplaines, IL	Wood waste and non-merchantable wood	Thermochemical gasification	Renewable gasoline	3,45,000	**Only demonstration scale**	Pilot scale/commercial production not started yet, gasoline manufactured at 3$ per gallon	8,9
Solazyme Inc. (Name changed to Terra Via™ on 11th March 2016)	Peoria, IL	Algae	Algae/biotechnology	Renewable hydrocarbons but switched to speciality chemicals	300,000	**Oprational but reported loss of 83 million $ last fiscal**	“We realized early on that we needed a business model that would allow us to use the power of our technology to produce any kind of microalgae and to enter higher-value markets,” says CEO. Margins on anti-aging serum are a heap better than what Solazyme can hope to glean from selling transport fuel (which costs roughly twice as much to produce than conventional diesel or jet fuel).	10,11
Archer Daniels Midland	Decatur, IL	Corn stover	Biochemical	Ethanol, butyl acrylate	25,800 ethanol, 21000 pound acrylate	**Operational with profits**	Low ethanol capacity. Wide range of products from beverages, chemicals to animal feed. Supplier of corn syrup to pepsico.	12,13
Myriant	Lake Providence, LA	Multifeedstock capacity	Biochemical	Succinic acid, lactic acid, fumaric acid, muconic acid, acrylic acid	30 million pound per year of succinic acid	**Operational with profits**	No fuel production. Target is only chemicals.	14,15

Verenium	Jennings, LA	Sugarcane bagasse, energy cane, sorghum	Biochemical	Ethanol	14,00,000	**Shutdown**	Demonstration plant acquired by BP in 2010	[16]
ICM Inc.	St. Joseph, MO	Switchgrass	Biochemical	Ethanol	2,45,000	**Operational with profits**	Changed feedstock from corn stover to switchgrass. Product range includes design and engineering/commissioning of unit operations related to biofuels	[17,18]
POET LLC	Emmetsburg, IA	Corn cobs	Biochemical	Ethanol and animal feed	20,000,000	**Operational without profit**	Unable to meet RFS demands, operations temporarily shutdown due to unavailability of corn	[19]
Abengoa Biorefinery	Hugoton, KS	Stover, switchgrass, woody biomass	Biochemical	Ethanol	25,000,000	**Shutdown**	Shutdown in Dec 2015	[20,21]
Rentech Clearfuels Technology	Commerce City, CO	Woody waste and bagasse	Thermochemical-Gasification	Renewable hydrocarbons	1,51,000	**Shutdown**	The plant and site of PSU have been opened for sale. Rentech reported loss of 119.9 million$ in 2015.	[22–24]
Sapphire Energy Inc	Columbus, NM	Algae	Algae	Renewable hydrocarbons	1,000,000	**Pilot scale**	Shifted focus from hydrocarbon fuels to nutraceuticals similar to approach of Solazyme Inc.	[25,26]
Zeachem Inc	Boardman, OR	Hybrid poplar, stover and cobs	Thermochemical-Gasification	Ethanol	2,50,000	**Demo scale**	Shifted focus from ethanol to chemicals and wood products. Ethanol only a side product now. Gasification shifted to biotechnology.	[27,28]

Logos Technologies	Visalia, CA	Corn stover, switchgrass, wood chips	Biochemical	Ethanol	50,000	**Pilot scale**	Shifted focus from ethanol. Company manufactures only biochemicals now.	[29,30]
Amyris Biotechnologies Inc.	Emeryville, CA	Sweet Sorghum	Biochemical	Renewable hydrocarbons	1370	**Operational but reported loss of 41.9 million $ in Q4 2016.**	Very low scale of renewable hydrocarbons. Wide product range.	[31,32]
UOP LLC	Kapolei, HI	Forest residues, corn stover, bagasse, switchgrass, algae	Thermochemical-pyrolysis	Renewable hydrocarbons	60,000	**Pilot scale shutdoen now**	Tesoro Corp. which was the source of hydrogen for the facility shutdown its operation resulting in cascade effect.	[33–35]
Algenol Biofuels Inc	Fort Meyers, FL	Algae	Algae	Ethanol	100,000	**Demo unit being explored for different technology**	Diversified in exploring CO2 capture and fresh water production. CEO & cofounder exits. 20% job cuts.	[36,37]
INEOS New Planet Bioenergy LLC	Vero Beach, FL	Vegetative and yard waste	Thermochemical-Gasification	Ethanol	8,000,000	**Shutdown**	Integrated Biorefinery Platform	[38,39]

References

1. *DOE/EE-0836 (January 2013)*.
2. Fishell, D. Creditors petition for old town fuel and fiber's bankruptcy claiming $1 million in debt. *Bangor Daily News* (2014).
3. *DOE/EE-0825 (December 2012)*.
4. *DOE/EE-0842 (January 2013)*.
5. *DOE/EE-0837 (January 2013)*.
6. Elevance Pilot-Scale Biorefinery. Available at: http://bit.ly/2bIXjgD.
7. Hussmann, P. Jasper County biorefinery project 'on hold indefinitely'. *Newton Independent* (2010).
8. *DOE/EE-0820 (December 2012)*.
9. Knight, R. *Green gasoline from wood using Carbona Gasification and Topsoe TIGAS Processes*. (2015).
10. Solazyme Pilot-Scale Biorefinery. Available at: http://bit.ly/2c1Zm39.
11. Helman, C. Forbes Disruptors 2013: Jonathan Wolfson Of Algae-Innovator Solazyme. *Forbes* (2013). Available at: http://bit.ly/2cg7mOE. (Accessed: 31st August 2016)
12. *DOE/EE-0813 (December 2012)*.
13. ADM: Products & Services. Available at: http://bit.ly/2bKKrIn.
14. *DOE/EE-0824 (December 2012)*.
15. Myriant: Products. Available at: http://bit.ly/2bElobb.
16. Tobben, S. BP said planning to close US cellulosic operation by end-1Q 2015. *Bloomberg Business* (2014).
17. *DOE/EE-0821 (December 2012)*.
18. ICM: Advanced Oil Separation System. Available at: http://bit.ly/2bDcnAH.
19. *DOE/EE-0827 (December 2012)*.
20. Abengoa Biorefinery. Available at: http://bit.ly/2ca9Omb.
21. Austin, S. Stevens County official: Abengoa plant shut down, workers laid off. *Telegram* (2015).

22. Rentech Integrated Biorefinery Pilot Project. Available at: http://bit.ly/2caa0lH.

23. Lane, J. Rentech to close Colorado demo unit, drop advanced biofuels R&D activities. *Biofuels Digest* (2013).

24. Rentech quaterly SEC filings. Available at: http://bit.ly/2c6fQFQ.

25. *DOE/EE-0841 (January 2013)*.

26. Bigelow, B. Algal biofuel icon Sapphire Energy moves to diversify product line. *Xconomy* (2015).

27. *DOE/EE-0822 (December 2012)*.

28. ZeaChem: Project Development. Available at: http://bit.ly/2c6h83L.

29. *DOE/EE-0823 (December 2012)*.

30. Schwab, A., Warner, E. & Lewis, J. *2015 Survey of Non-Starch Ethanol and Renewable Hydrocarbon Biofuels Producers*. (2015).

31. *DOE/EE-0814 (December 2012)*.

32. Amyris reports 4Q loss. *CNBC* (2016).

33. *DOE/EE-0826 (December 2012)*.

34. Wissinger, R. *Pilot-Scale Biorefinery: Sustainable Transport Fuels from Biomass via Integrated Pyrolysis, Catalytic Hydroconversion and Co-processing with Vacuum Gas Oil*. (2015).

35. Daysog, R. Tesoro Hawaii refinery to shut down, 200 jobs to be lost. *Hawaii News Now* (2013).

36. *DOE/EE-0835 (January 2013)*.

37. Lane, J. Algenol CEO exits; staff cut by 25%, investors re-up for two years, new direction tipped. *Biofuels Digest* (2015).

38. Russell, K. 2015 DOE IBR Platform Peer Review. Available at: http://bit.ly/2bL7PFA.

39. Baita, S. INEOS bio plant shut; produced excess deadly gas. *VeroNews* (2015).
